# Poincaré plot can help predict the curative effect of metoprolol for pediatric postural orthostatic tachycardia syndrome

**DOI:** 10.3389/fnins.2023.1280172

**Published:** 2023-11-14

**Authors:** Piaoliu Yuan, Zhouhui Lian, Yuanyuan Wang, Chunyu Zhang, Hongfang Jin, Junbao Du, Yaqian Huang, Ying Liao

**Affiliations:** ^1^Department of Pediatrics, Peking University First Hospital, Beijing, China; ^2^Department of Pediatrics, The First Affiliated Hospital of Guangxi Medical University, Nanning, Guangxi, China; ^3^Wang Xuan Institute of Computer Science, Peking University, Beijing, China; ^4^State Key Laboratory of Vascular Homeostasis and Remodeling, Peking University, Beijing, China

**Keywords:** Poincaré plot, metoprolol, therapeutic outcome, children, postural orthostatic tachycardia syndrome

## Abstract

**Purpose:**

To study whether a Poincaré plot can help predict the curative effect of metoprolol for postural orthostatic tachycardia syndrome (POTS) in children.

**Methods:**

Pediatric patients with POTS who were administered metoprolol were retrospectively included. The collected data included general data (sex, age, height, weight, and body mass index), the manifestations and treatment (baseline orthostatic intolerance symptom score and course of metoprolol treatment), vital signs (supine heart rate [HR], supine blood pressure, and increased HR during the standing test), HR variability indexes (standard deviation of normal-to-normal intervals [SDNN]; standard deviation of the averages of normal-to-normal intervals [SDANN]; mean standard deviation of the NN intervals for each 5-min segment [SDNNI]; root mean square of the successive differences [rMSSD]; percentage of adjacent NN intervals that differ by >50 ms [pNN50]; triangular index; ultra-low [ULF], very low [VLF], low [LF], and high frequency [HF]; total power [TP]; and LF/HF ratio), and graphical parameters of the Poincaré plot (longitudinal axis [L], transverse axis [T], and L/T). Receiver operator characteristic curves were used to calculate the predictive function of the indexes with significant differences between patients who responded and those who did not. The index combination with the highest predictive value was obtained through series–parallel analysis.

**Results:**

Overall, 40 responders and 23 non-responders were included. The L and T in the Poincaré plots and rMSSD, pNN50, HF, and TP of the HR variability data were significantly lower in participants who responded to metoprolol than in participants who did not (*p* < 0.001). The L/T of participants who responded to metoprolol was greater than that of non-responders (*p* < 0.001). Moreover, we noted a strong correlation between every two indexes among L, T, rMSSD, pNN50, HF, TP, and L/T (*p* < 0.05). T < 573.9 ms combined with L/T > 2.9 had the best performance for predicting the effectiveness of metoprolol, with a sensitivity of 85.0%, specificity of 82.6%, and accuracy of 84.1%.

**Conclusion:**

In the Poincaré plot, a T < 573.9 ms combined with an L/T > 2.9 helps predict good outcomes of using metoprolol to treat pediatric POTS.

## Introduction

Pediatric orthostatic intolerance (OI), a clinical condition that can be relieved by lying down, typically after standing upright, can manifest as dizziness, cephalalgia, nausea, vomiting, chest distress, cardiopalmus, blurred vision, and even syncope (Stewart, 2002). OI most commonly occurs in school-aged children and adolescents and can affect their daily activities and attendance (Boris, 2018; Wang et al., 2018). Postural orthostatic tachycardia syndrome (POTS) is the primary cause of chronic OI in children (Stewart, 2013; Lin et al., 2016; Wang et al., 2018), for which metoprolol is most commonly prescribed (Chen et al., 2020; Wang et al., 2021). Metoprolol has been used indiscriminately in children diagnosed with POTS, resulting in distinct therapeutic effects. Only 57–63.6% of patients responded to metoprolol (Lai et al., 2009; Chen et al., 2011), possibly owing to the diversity and complexity of the pathogenesis of POTS (Boris, 2018; Wells et al., 2018; Chen et al., 2020). Currently, the pathogenesis of POTS includes central hypovolemia, hyperadrenergic state, decreased blood pumping function of skeletal muscles, abnormal local vasoconstriction function, and abnormal vasoactive factor release (Boris, 2018; Wells et al., 2018; Chen et al., 2020). Metoprolol acts as a blocker by antagonizing adrenergic receptor hyperactivation and has been speculated to work mainly in children with a hyperadrenergic state. Therefore, biomarkers are needed to identify children with a high adrenergic status in clinical practice to individualize the therapeutic regimen and improve the efficacy of pediatric POTS management. Previous studies have used plasma noradrenaline (Zhang et al., 2014), plasma copeptin (Zhao et al., 2014), C-type natriuretic peptide (CNP) (Zhao et al., 2014), and indexes of heart rate (HR) variability (Wang et al., 2019) to identify children with POTS for whom metoprolol could be effective. However, the plasma level of noradrenaline is easily affected by emotional factors, such as emotional stress and agitation, as well as pathological factors, such as the presence of an adrenal adenoma and pheochromocytoma. Additionally, the laboratory test for plasma copeptin and CNP requires blood to be drawn from children, and many clinical facilities cannot perform these tests, which may lead to popularization difficulties. Moreover, the predictive efficiency of the time domain index of HR variability needs to be improved. Therefore, we urgently need to find stable, easy-to-apply, and intuitive predictors to guide metoprolol administration.

Children with POTS usually have symptoms such as chest tightness or palpitations and require Holter electrocardiogram (ECG) monitoring to rule out potential arrhythmias. Poincaré plots are graphics derived from long-term (i.e., 24-h) ECG recordings (Carrasco et al., 2001; Naranjo Orellana et al., 2015) that have been widely used to diagnose arrhythmias, including identifying premature beats and diagnosing atrial fibrillation. In addition, Poincaré plots can intuitively reflect the comprehensive characteristics of the HR variability (Copie et al., 1996; Toichi et al., 1997), their shape can reflect the activity of the sympathetic and vagus nerves, and they are noninvasive, intuitive, and stable indicators of autonomic nervous function (Copie et al., 1996; Toichi et al., 1997). Based on this, we have previously reported in pediatric population that Poincaré plots can be used to distinguish vasovagal syncope (VVS) from POTS and predict the efficacy of metoprolol in the treatment of VVS (Yuan et al., 2022a,b). The mechanism of metoprolol treatment for POTS mainly involves addressing autonomic nervous function imbalance. In addition, Poincaré plots can be automatically generated by the Holter ECG monitoring equipment, which is noninvasive and intuitive and allows convenient access. Therefore, we speculated that Poincaré plots could be used to predict the clinical efficacy of metoprolol for POTS. Thus, we aimed to develop biomarkers derived from Poincaré plots to help predict the curative effect of metoprolol in pediatric POTS.

## Materials and methods

### Participants

Pediatric patients aged 6–18 years who were diagnosed with POTS between January 2012 and December 2020 at the Children’s Syncope Center of Peking University First Hospital and received metoprolol for more than 1 month were included in the study.

The exclusion criteria were as follows: (1) the course of OI symptoms was <3 months before hospitalization; (2) diagnosis of other diseases that could affect autonomic functions or Poincaré plots, such as arrhythmia, infection, hypertension, diabetes, anemia, and thyroid dysfunction; (3) concurrent receipt of medications other than metoprolol that could affect autonomic functions or Poincaré plots; (4) not taking metoprolol as directed or having contraindications for metoprolol (i.e., sinus bradycardia, atrial-ventricular block, asthma, and allergy to metoprolol) (Wang et al., 2018); and (5) incomplete clinical data or poor Poincaré plot quality.

We collected data from each participant by searching the Medical Record System (Kaihua Medical Recording Management Digital System). The collected data included general data (sex, age, height, weight, and body mass index), the manifestations and treatment (baseline [i.e., the time of diagnosis] OI symptom score and course of metoprolol treatment), vital signs (supine HR, supine systolic blood pressure [BP], supine diastolic BP, increased HR during standing test [the maximum difference between upright HR and recumbent HR during standing test]), HR variability indexes (standard deviation of normal-to-normal intervals [SDNN]; standard deviation of the averages of normal-to-normal intervals [SDANN]; mean standard deviation of the NN intervals for each 5-min segment [SDNNI]; root mean square of the successive differences [rMSSD]; percentage of adjacent NN intervals that differ by >50 ms [pNN50]; triangular index; ultra-low [ULF], very low [VLF], low [LF], and high frequency [HF]; total power [TP]; and LF/HF ratio), and graphical parameters of the Poincaré plot (longitudinal axis [L], transverse axis [T], and L/T ratio).

This study complied with the Declaration of Helsinki and was approved by the Ethics Committee of Peking University First Hospital (2021 [150]). Informed consent was obtained from the legal guardians of the patients.

### Diagnosis of POTS

POTS was diagnosed based on the following criteria: (1) triggers such as constant standing or rapid postural changes; (2) corresponding symptoms (e.g., dizziness, cephalalgia, lassitude, blurring of vision, chest distress, cardiopalmus, hand tremors, physical decline, and syncope); (3) a positive response that indicated POTS in the standing test or the initial 10 min of the head-up tilt test (HUTT); and (4) other diseases causing similar symptoms of OI were ruled out. The positive criteria for POTS in the associated tests included the following: (1) normal HR when lying on the back; (2) within the first 10 min after standing up in standing test or tilted in HUTT an increase in HR (≥ 40 bpm) and/or a maximum HR ≥ 130 and ≥ 125 bpm for ages 6–12 and 13–18 years, respectively; (3) with out significant orthostatic hypotension (decline of BP < 20/10 mmHg) (Medow et al., 2017; Wang et al., 2018).

### OI symptom score

The severity of POTS and the curative effect of metoprolol were evaluated using the OI symptom score, which was based on the following 10 major OI symptoms: syncope, dizziness, cephalalgia, chest distress, cardiopalmus, nausea, tremor, sweating, blurred vision, and lack of concentration (Li et al., 2016; Wang et al., 2019). To obtain the OI symptom score, the scores for each of the above symptoms were summed, based on the average frequency of OI symptoms before treatment and during follow-up: 0, no such symptoms; 1 point, no more than once/month; 2 points, more than once/month but no more than once/week; 3 points, more than once/week but no more than once/day; and 4 points, more than once/day (Li et al., 2016; Wang et al., 2019).

### Standing test and HUTT

#### Standing test

Participants were required to lay on a bed for 10–30 min and then stand upright for 10 min. An ECG monitor (Dash 2000; General Electric, New York, USA) recorded the HR, BP, and ECG without interruption (Wang et al., 2018). All children received two to three standing tests during hospitalization and the highest increase of HR were recorded.

#### HUTT

Although the standing test is relatively simple and should be prioritized in clinical practice, the HUTT can also be used to help diagnose of POTS. Before starting, the children were informed of the need to abstain from food and water for 4 h and not to take medications affecting the autonomic tone and cardiovascular function. To begin the HUTT, participants were required to lay quietly on the examining bed (SHUT 100A Standley and Hut 821, Beijing Juchi Medical Technology Co., Ltd., Beijing, China) for approximately 10–30 min. The examination room was kept warm, quiet, and dimly lit. We used a multi-guide ECG (Dash 2000; General Electric, New York, USA) to record the HR, BP, and ECG. After tilting the bed to 60°, the patient stood passively on the tilted bed, and the above hemodynamic parameters were continuously monitored. If the child had a positive reaction, the test was terminated, and the examination bed was quickly returned to the horizontal position. Otherwise, the test was continued for 45 min (Wang et al., 2018).

### Holter ECG monitoring and Poincaré plot parameters

Twenty-four-hour Holter examination: The day before and on the day of the examination, the children were asked to avoid food or drink (e.g., coffee, tea, and alcohol) and medications that could affect sympathetic and vagal nerve activity and avoid prolonged strenuous exercise and emotional agitation. The 24-h Holter ECG data were monitored and analyzed using the H3 + ™/H12 + ™ Mortara Holter ECG and H-Scribe 7.0, respectively (Mortara Instrument, Milwaukee, Wisconsin, USA). Poincaré plots were automatically constructed.

**Figure 1:**
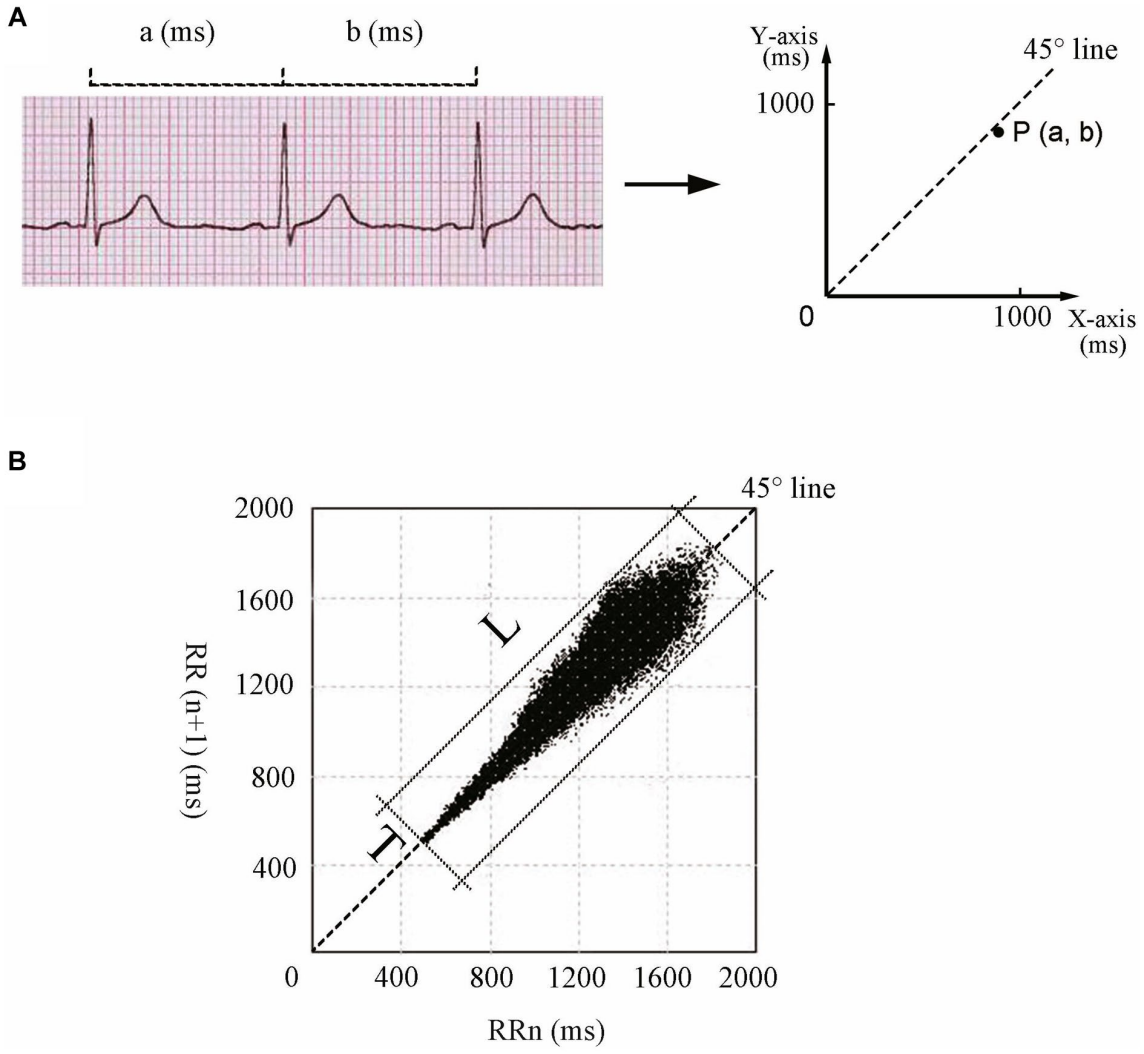
Construction and parameters of Poincaré plots. **(A)** Construction principle of Poincaré plots. Each dot in the Poincaré plot is determined by the duration of a heartbeat interval (a, ms) as the horizontal coordinate and the duration of the next heartbeat interval (b, ms) as the vertical coordinate. **(B)** L and T of the Poincaré plot for an 11-year-old girl. L of the Poincaré plot is the longest straight distance of the graph distributed along the 45° line, and T is the distance between the two straight lines parallel to the 45° line intersecting the dense part of the graph boundary. L, longitudinal axis; T, transverse axis.

Figure 1A illustrates the construction of Poincaré plots. A point was obtained in the coordinate system by taking the duration of one heartbeat interval (ms) as the horizontal coordinate, and the duration of the next heartbeat interval (ms) as the vertical coordinate. Therefore, a point in the Poincaré plot was obtained for every three QRS (R) waves or two RR intervals. Scatter points with the same characteristics were clustered, forming the Poincaré plot of the Holter ECG data (Keeley et al., 1997). A Poincaré plot was constructed for each 24-h Holter ECG data set. The Poincaré plot is a statistical map of cardiac cycles. L denotes the length of the graph distributed along the 45° line, and T is the vertical distance between the two lines parallel to the 45° line and tangential to the dense part of the graph boundary (Brennan et al., 2002; Ueda et al., 2002; Chaidas et al., 2014; Figure 1B). Following the generation of all Poincaré plots, we used plotting software (Adobe Photoshop 2020, SAN Jose, California, USA) to measure the graphical parameters L and T (Figure 1B). L/T was calculated from L and T values. A specially assigned person measured the graphs using a method previously shown to have good stability and repeatability (Yuan et al., 2022a,b). The method and results of the stability and repeatability tests can be found in the Supplementary material.

### Analysis of HR variability

The 12-lead 24-h Holter ECG monitor (H3 + ™/H12 + ™, Mortara Instrument, Milwaukee, Wisconsin, USA) used to obtain Poincaré plots was also used to examine the HR variability parameters. The sample frequency was 10,000 Hz, and the frequency response range was 0.05–60 Hz. The HR variability was assessed using an analyzer (H-Scribe 7.0; Mortara Instruments, Milwaukee, Wisconsin, USA). The first step in HR variability analysis is to identify the R-waves on the ECG waveform and thus determine the cardiac cycles. The Holter ECG analysis system (Mortara, H-Scribe 7.0 Holter analysis system) checks the signal quality on each beat to determine the best quality channels to be used. Beat detection channels may change dynamically as the analysis progresses. Intermittent periods of lead fail or artifact will cause analysis channel switching to ensure accurate beat labeling. Signal processing is performed to remove or lessen the types of noise and artifacts that occur during ambulatory recording. The computer-analyzed recordings were reviewed by two qualified clinicians to ensure a highly accurate final report. Each RR interval was examined to exclude ectopic HRs such as ventricular or supraventricular rhythms. Therefore, only normal-to-normal RR intervals were analyzed, and cases with an inadequate Holter ECG recording time (< 20 h) were excluded. Each segment analyzed in the time domain was composed of >50% of the normal RR interval, and each segment analyzed in the frequency domain was composed of >80% of the normal RR interval. The time-domain indexes of HR variability included SDNN, SDANN, SDNNI, rMSSD, pNN50, and the triangular index, while the frequency-domain indexes included ULF, VLF, LF, HF, TP, and LF/HF (Wang et al., 2019). The frequency-domain indexes were calculated by the spectrum analysis of the Fast Fourier Transform method. Records with frequencies >60 Hz were de-trended and low-pass filtered for removal. The power of frequency bands could be divided into four bands: ULF (0–0.003 Hz), VLF (0.003–0.04 Hz), LF (0.04–0.15 Hz), and HF (0.15–0.40 Hz). TP was the total power of NN interval change within 5 min (0–0.4 Hz), and LF/HF was the ratio of LF to HF.

### Treatment with metoprolol and therapeutic effect evaluation

All enrolled participants received general treatment (i.e., health education, avoidance of triggers, increased water and salt intake, and physical compression maneuvers). The oral dose of metoprolol (AstraZeneca, London, United Kingdom) for all enrolled children with POTS was 0.5 mg/kg/day, with dosage increases as required but not exceeding 2 mg/kg (up to 50 mg) daily. Metoprolol was discontinued if the HR fell below 60 bpm at rest.

Baseline OI symptom scores were calculated before management with metoprolol, and follow-up started after the initial admission, during which POTS was first diagnosed. Participants were re-evaluated through rehospitalization, outpatient visits, or telephone interviews. Compliance with the therapy was recorded, and OI symptom scores were recalculated after 3 months of treatment with metoprolol. Participants were considered responders if the recalculated OI symptom scores decreased by at least two points compared with the baseline OI symptom scores. Otherwise, the participants were considered non-responders (Li et al., 2016; Wang et al., 2019).

### Statistical analysis

SPSS 21.0 (IBM, New York, America) was used to conduct all statistical analyses. Normality was tested using the Shapiro–Wilk test. Normally distributed data are expressed as the mean ± standard deviation, and the independent sample *t* test was used to compare data between the two groups. Non-normally distributed data are expressed as the median (interquartile range [IQR], [P25, P75]), and the Mann–Whitney *U* nonparametric test was used to compare data between the two groups. Categorical data are expressed as *n* (%) and were compared between groups using the chi-square test. Spearman’s linear correlation analysis was used to analyze the correlations. A receiver operating characteristic (ROC) curve was applied to evaluate whether the indexes helped predict the therapeutic outcome of metoprolol. The statistics were the area under the curve, 95% confidence interval, and *p*-value. Differences with *p*-values <0.05 were considered significant.

## Results

### Clinical data of responders and non-responders for metoprolol therapy

**Figure 2:**
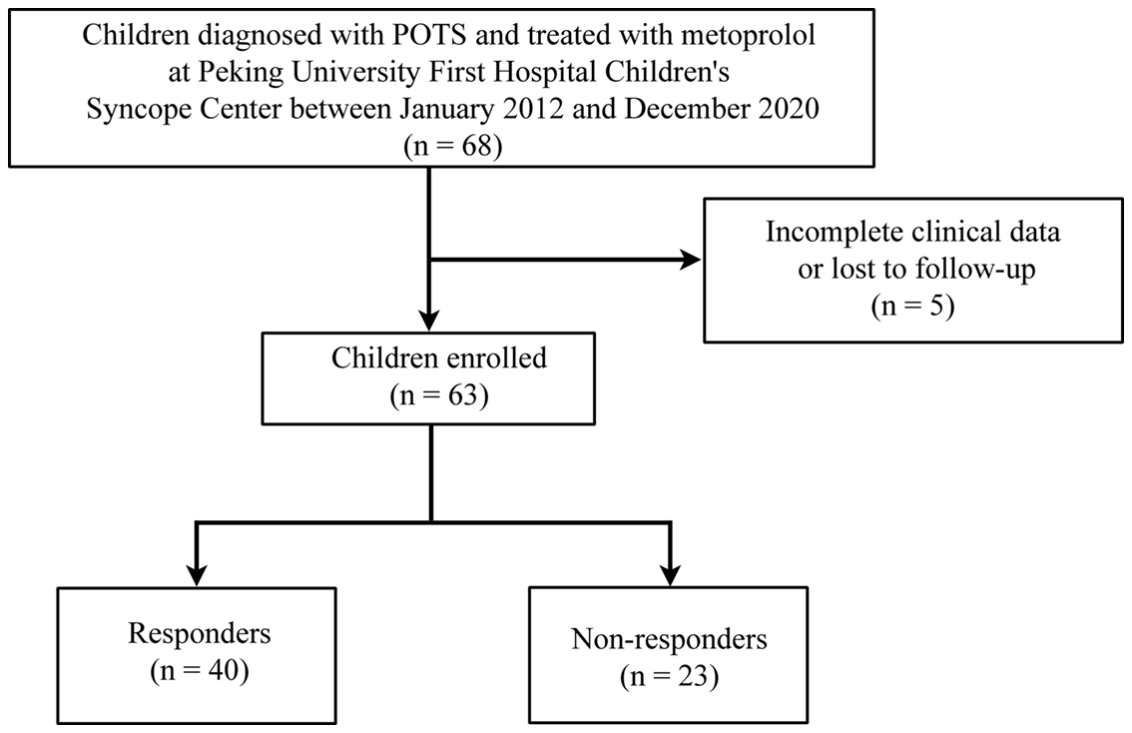
Flowchart of inclusion and exclusion of participants. POTS, postural orthostatic tachycardia syndrome.

A total of 63 children with POTS (36 boys and 27 girls; median age: 12.0 years; range: 7.0–16.0 years), including 40 responders (21 boys and 19 girls; median age: 12.0 years; range: 7.0–16.0 years) and 23 non-responders (15 boys and 8 girls; median age: 13.0 years; range: 8.0–16.0 years), were analyzed. The results of the standing test performed by all 63 children were positive for POTS. Of the 51 children who underwent the HUTT, only 26 had positive POTS results. Figure 2 shows a flowchart of the selection of patients for this study. The L, T, rMSSD, pNN50, HF, and TP of the responders were significantly lower than those of the non-responders (*p* < 0.05), and the L/T and LF/HF of the responders were significantly higher than those of the non-responders (*p* < 0.05) (Table 1).

**Table 1:** Clinical data of patients who did and did not respond to metoprolol treatment for POTS.

Variables	Responders (*n* = 40)	Non-responders (*n* = 23)	t/Z/χ^2^	*P*
Sex, male/female	21/19	15/8	0.964	0.326
Age, years	12.4 ± 2.1	12.3 ± 1.8	−0.135	0.893
Height, cm	159.7 ± 13.9	162.4 ± 8.4	0.964	0.339
Weight, kg	53.5 (39.1–60.0)	52.5 (42.8–60.5)	−0.029	0.977
BMI, kg/m^2^	18.9 (17.1–23.1)	19.5 (18.2–22.8)	−0.671	0.502
Baseline OI symptom score, points	6.0 (4.0–8.8)	8.0 (5.0–12.0)	−1.559	0.119
Metoprolol treatment, months	2.0 (1.0–3.0)	2.0 (1.0–3.0)	−0.300	0.764
HR, bpm	75.5 (70.5–84.5)	45.0 (43.0–50.0)	0.215	0.830
Systolic pressure, mmHg	109.0 (102.0–119.8)	113.0 (110.0–117.0)	−1.307	0.191
Diastolic pressure, mmHg	60.5 (55.3–68.8)	66.0 (59.0–70.0)	−1.708	0.088
Maximum heart rate during standing test, bpm	124.0 (118.0–133.0)	123.0 (117.0–133.0)	−0.222	0.825
Increased heart rate during standing test^a^, bpm	45.5 (42.0–52.0)	76.0 (69.0–83.0)	−0.215	0.830
SDNN, ms	136.5 ± 34.8	153.6 ± 34.9	1.872	0.066
Triangular index	28.0 (23.3–32.5)	34.0 (27.0–38.0)	−1.873	0.061
SDNNI	69.1 ± 25.0	77.8 ± 20.5	1.424	0.160
rMSSD, ms	44.3 ± 14.9	59.8 ± 24.1	2.793	0.009
pNN50, %	18.2 ± 10.0	25.1 ± 12.7	2.383	0.020
SDANN, ms	123.5 (100.3–154.5)	143.0 (111.0–152.0)	−1.506	0.132
ULF, ms^2^	11,449.5 (8,767.9–18,367.3)	17,943.5 (9,622.5–22,780.7)	−1.613	0.107
VLF, ms^2^	2,142.1 (1,677.6–3,016.3)	2,955.5 (1,922.3–4,296.0)	−1.799	0.072
LF, ms^2^	881.4 (622.9–1,205.4)	1,139.3 (790.7–1,846.8)	−1.942	0.052
HF, ms^2^	577.5 (382.7–937.0)	1,239.4 (289.0–1,864.3)	−2.470	0.014
TP, ms^2^	3,017.6 (1,880.2–3,510.2)	4,410.0 (2,238.5–5,910.5)	−2.313	0.021
LF/HF	1.4 (1.2–1.8)	1.2 (0.8–1.7)	−2.063	0.039
L, ms	1,502.6 ± 225.2	1,733.7 ± 231.9	3.880	< 0.001
T, ms	432.7 ± 122.3	705.3 ± 201.2	5.901	< 0.001
L/T	3.6 (3.1–4.1)	2.4 (2.2–3.0)	−4.683	< 0.001

*p* < 0.05, statistically significant. POTS, postural orthostatic tachycardia syndrome; OI, orthostatic intolerance; HR, heart rate; bpm, beat per minute; SDNN, standard deviation of normal-to-normal intervals; SDNNI, standard deviation of the averages of NN intervals; rMSSD, root mean square of the successive differences; pNN50, percentage of adjacent NN intervals that differed by > 50 ms; SDANN, standard deviation of the averages of normal-to-normal intervals in all 5-min segments of the entire recording; ULF, ultralow frequency; VLF, very low frequency; LF, low frequency; HF, high frequency; TP, total power; L, longitudinal axis; T, transverse axis. ^a^The maximum difference between upright and recumbent heart rates during the standing test.

### Predictive value of the Poincaré plot and HR variability in response to metoprolol

**Figure 3:**
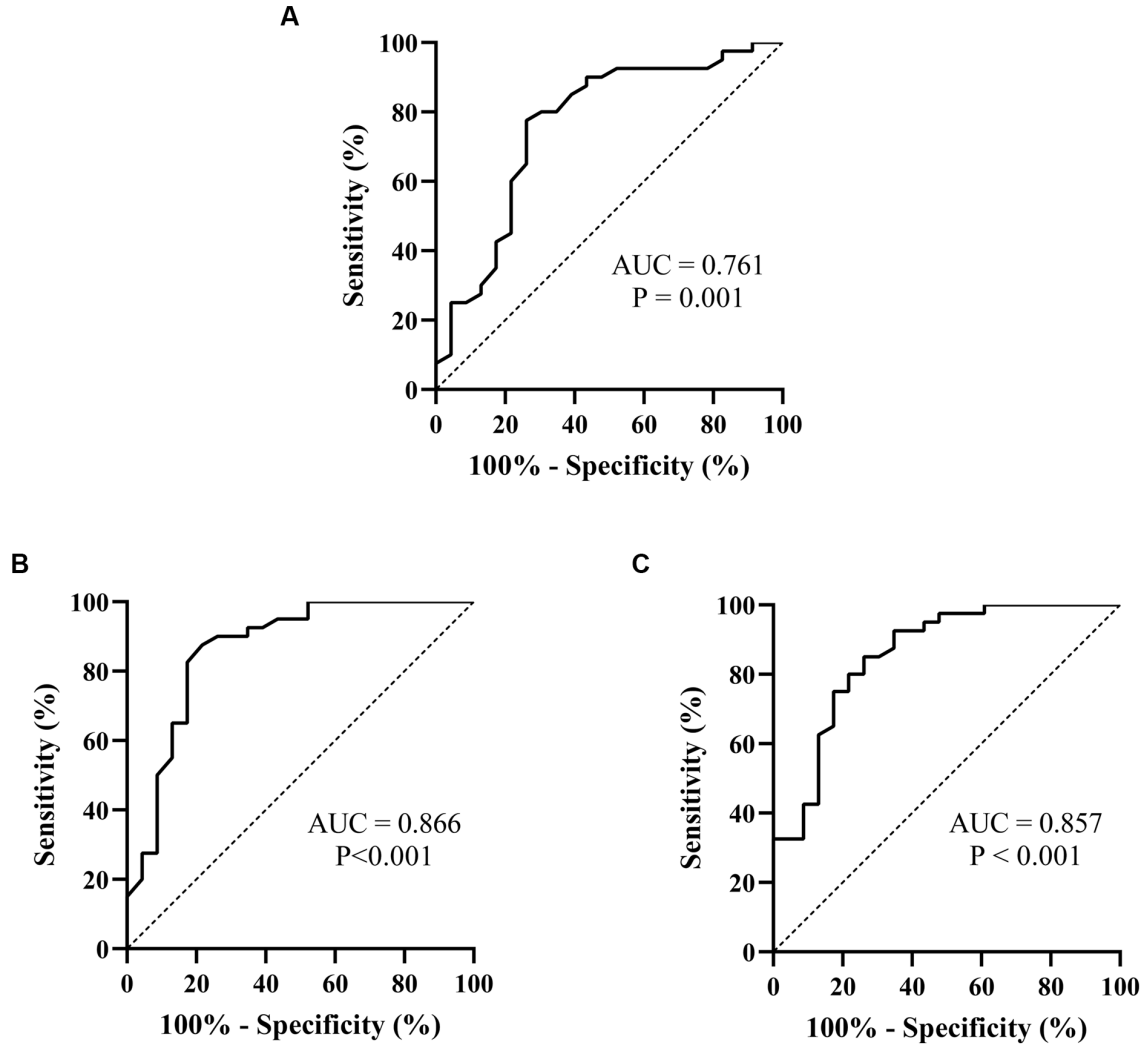
ROC curves of the Poincaré plot indexes for prejudging the outcome of metoprolol in treating pediatric POTS. **(A)** L; **(B)** T; **(C)** L/T. The ordinate represents the sensitivity to prejudge the treatment outcome of metoprolol, and the abscissa shows the false-positive rate (i.e., 100% − specificity). The sensitivity of the 45° line in the figure is equal to the false-positive rate, meaning no effect for prejudging. AUC, area under the curve; ROC, receiver operating characteristic; POTS, postural orthostatic tachycardia syndrome; L, longitudinal axis; T, transverse axis.

**Figure 4:**
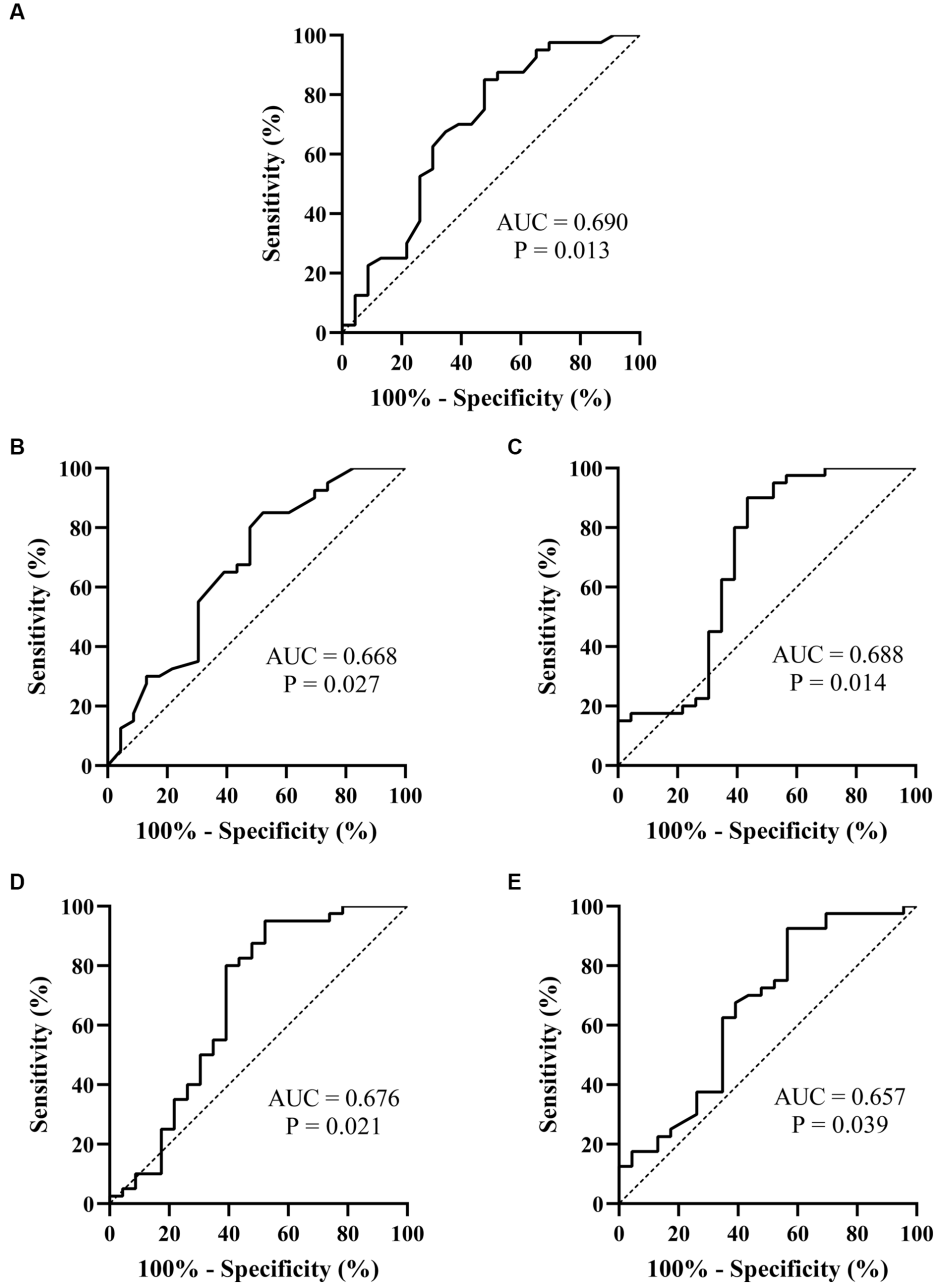
ROC curves of the heart rate variability indexes for prejudging the treatment outcome of metoprolol for pediatric POTS. **(A)** rMSSD; **(B)** pNN50; **(C)** HF; **(D)** TP; **(E)** LF/HF. The ordinate represents the sensitivity to prejudge the treatment outcome of metoprolol, and the abscissa shows the false positive rate (i.e., 100% − specificity). The sensitivity of the 45° line in the figure is equal to the false positive rate, meaning no effect for prejudging. POTS, postural orthostatic tachycardia syndrome; AUC, area under the curve; ROC, receiver operating characteristic; rMSSD, root mean square of the successive difference; pNN50, percentage of adjacent NN intervals that differ by >50 ms; HF, high frequency; TP, total power; LF, low frequency.

Spearman’s linear correlation analysis was performed on the indexes (L, T, rMSSD, pNN50, HF, TP, L/T, and LF/HF) that were significantly different between patients who did and did not respond to metoprolol. A strong correlation was found between every two indexes, including L, T, rMSSD, pNN50, HF, TP, L/T, and LF/HF (*p* < 0.05). The ROC analysis found the above parameters of the Poincaré plot (Table 2; Figure 3) and the HR variability indexes (Table 2; Figure 4) significantly predicted the treatment outcome of using metoprolol (*p* < 0.05).

**Table 2:** Predictive value of the parameters on the efficacy of metoprolol treatment.

Variables	AUC	*P*	95% CI	Cut-off value	Sensitivity (%)	Specificity (%)
L	0.761	0.001	0.630–0.893	1,680.7 ms	77.5	73.9
T	0.866	< 0.001	0.764–0.969	573.9 ms	87.5	78.3
L/T	0.857	< 0.001	0.757–0.956	2.9	85.0	73.9
rMSSD	0.690	0.013	0.544–0.835	59.5 ms	85.0	52.2
pNN50	0.668	0.027	0.523–0.814	27.5 ms	85.0	47.8
HF	0.688	0.014	0.532–0.844	1,144.0 ms^2^	90.0	56.5
TP	0.676	0.021	0.520–0.832	4,740.7 ms^2^	95.0	47.8
LF/HF	0.657	0.039	0.509–0.806	1.1	92.5	43.5

*p* < 0.05, statistically significant. AUC, area under the curve; CI, confidence interval; L, longitudinal axis; T, transverse axis; rMSSD, root mean square of successive differences; pNN50, percentage of adjacent NN intervals that differed by > 50 ms; HF, high frequency; TP, total power; LF, low frequency.

### Performance of combined indexes for predicting the therapeutic outcome of metoprolol

Because of the high correlation between the above parameters, the above indexes (L, T, L/T, rMSSD, pNN50, HF, TP, and LF/HF) were analyzed in series and parallel to obtain the optimal prediction efficiency (Table 3). The results showed that a T < 573.9 ms combined with an L/T > 2.9 had the highest value in predicting the treatment outcome of using metoprolol (sensitivity: 85.0%; specificity: 82.6%; accuracy: 84.1%).

**Table 3:** Series–parallel analysis to prejudge the outcome of metoprolol treatment for pediatric POTS.

Indexes of series–parallel analysis	Sensitivity (%)	Specificity (%)	Accuracy (%)
T < 573.9 ms combined with L/T > 2.9	85.0	82.6	84.1
T < 573.9 ms or L/T > 2.9	87.5	69.6	81.0
T < 573.9 ms combined with L < 1,680.7 ms	77.5	73.9	76.2
T < 573.9 ms or L < 1,680.7 ms	92.5	69.6	84.1
T < 573.9 ms combined with rMSSD <59.5 ms	80.0	78.3	79.4
T < 573.9 ms or rMSSD <59.5 ms	95.0	52.2	79.4
T < 573.9 ms combined with pNN50 < 27.5 ms	75.0	78.3	76.2
T < 573.9 ms or pNN50 < 27.5 ms	97.5	47.8	79.4
T < 573.9 ms combined with HF < 1,144.0 ms^2^	87.5	78.3	84.1
T < 573.9 ms or HF < 1,144.0 ms^2^	95.0	56.5	81.0
T < 573.9 ms combined with TP < 4,740.7 ms^2^	82.5	78.3	81.0
T < 573.9 ms or TP < 4,740.7 ms^2^	100	47.8	81.0
T < 573.9 ms combined with LF/HF > 1.1	80.0	78.3	79.4
T < 573.9 ms or LF/HF > 1.1	97.5	43.5	77.8
L < 1,680.7 ms combined with L/T > 2.9	70.0	87.0	76.2
L < 1,680.7 ms or L/T > 2.9	100.0	60.9	85.7
L < 1,680.7 ms combined with rMSSD <59.5 ms	72.5	69.6	71.4
L < 1,680.7 ms or rMSSD <59.5 ms	72.5	52.2	65.1
L < 1,680.7 ms combined with pNN50 < 27.5 ms	72.5	69.6	71.4
L < 1,680.7 ms or pNN50 < 27.5 ms	92.5	47.9	76.2
L < 1,680.7 ms combined with HF < 1,144.0 ms^2^	82.5	73.9	79.4
L < 1,680.7 ms or HF < 1,144.0 ms^2^	90.0	56.5	77.8
L < 1,680.7 ms combined with TP < 4,740.7 ms^2^	77.5	73.9	76.2
L < 1,680.7 ms or TP < 4,740.7 ms^2^	95.0	47.8	77.8
L < 1,680.7 ms combined with LF/HF > 1.1	75.0	73.9	74.6
L < 1,680.7 ms or LF/HF > 1.1	95.0	43.5	76.2
L/T > 2.9 combined with rMSSD <59.5 ms	75.0	73.9	74.6
L/T > 2.9 or rMSSD <59.5 ms	95.0	52.2	79.4
L/T > 2.9 combined with pNN50 < 27.5 ms	72.5	73.9	73.0
L/T > 2.9 or pNN50 < 27.5 ms	97.5	47.8	79.4
L/T > 2.9 combined with HF < 1,144.0 ms^2^	82.5	73.9	79.4
L/T > 2.9 or HF < 1,144.0 ms^2^	95.0	56.5	81.0
L/T > 2.9 combined with TP < 4,740.7 ms^2^	80.0	78.3	79.4
L/T > 2.9 or TP < 4,740.7 ms^2^	100.0	43.5	79.4
rMSSD <59.5 ms combined with pNN50 < 27.5 ms	82.5	52.2	71.4
rMSSD <59.5 ms or pNN50 < 27.5 ms	87.5	47.8	73.0
rMSSD <59.5 ms combined with HF < 1,144.0 ms^2^	85.0	60.9	76.2
rMSSD <59.5 ms or HF < 1,144.0 ms^2^	90.0	47.8	74.6
rMSSD <59.5 ms combined with TP < 4,740.7 ms^2^	85.0	56.5	74.6
rMSSD <59.5 ms or TP < 4,740.7 ms^2^	95.0	43.5	76.2
rMSSD <59.5 ms combined with LF/HF > 1.1	77.5	60.9	71.4
rMSSD <59.5 ms or LF/HF > 1.1	97.5	34.8	74.6
pNN50 < 27.5 ms combined with HF < 1,144.0 ms^2^	77.5	65.2	73.0
pNN50 < 27.5 ms or HF < 1,144.0 ms^2^	92.5	43.5	74.6
pNN50 < 27.5 ms combined with TP < 4,740.7 ms^2^	85.0	56.5	74.6
pNN50 < 27.5 ms or TP < 4,740.7 ms^2^	95.0	39.1	74.6
pNN50 < 27.5 ms combined with LF/HF > 1.1	77.5	60.9	71.4
pNN50 < 27.5 ms or LF/HF > 1.1	97.5	30.4	73.0
HF < 1,144.0 ms^2^ combined with TP < 4,740.7 ms^2^	90.0	60.9	79.4
HF < 1,144.0 ms^2^ or TP < 4,740.7 ms^2^	95.0	43.5	76.2
HF < 1,144.0 ms^2^ combined with LF/HF > 1.1	82.5	56.5	73.0
HF < 1,144.0 ms^2^ combined with TP < 4,740.7 ms^2^	90.0	60.9	79.4
HF < 1,144.0 ms^2^ or LF/HF > 1.1	97.5	43.5	77.8
TP < 4,740.7 ms^2^ combined with LF/HF > 1.1	85.0	60.9	76.2
TP < 4,740.7 ms^2^ or LF/HF > 1.1	100.0	30.4	74.6

L, longitudinal axis; T, transverse axis; rMSSD, root mean square of successive differences; pNN50, percentage of adjacent NN intervals that differed by > 50 ms; HF, high frequency; TP, total power; LF, low frequency.

## Discussion

We innovatively proved that the L and T from the Poincaré plots, as well as rMSSD, pNN50, HF, and TP values for children who responded to metoprolol, were significantly smaller than those of children who did not respond to metoprolol for pediatric POTS. In contrast, the L/T and LF/HF of the children who responded to metoprolol were observably larger than those of children who did not. A series–parallel analysis revealed a high efficiency (sensitivity: 85.0%; specificity: 82.6%; accuracy: 84.1%), indicating a curative outcome for metoprolol treatment for pediatric POTS with a combination of T < 573.9 ms and L/T > 2.9. We demonstrated for the first time that Poincaré plots can be used as a conveniently accessed, noninvasive, and intuitive method to predict the efficacy of using metoprolol for POTS.

POTS has the highest incidence in pediatric patients with chronic OI (Spahic et al., 2019). Metoprolol, a selective β1 adrenergic receptor-blocker, has been reported to be effective in pediatric POTS (Bryarly et al., 2019). However, when β-blockers are used indiscriminately, the effectivity rate is only approximately 57–63.6% (Lai et al., 2009; Chen et al., 2011). Additionally, metoprolol has potential side effects when misused, such as hypotension, bradycardia, atrioventricular block, fatigue, and reduced exercise endurance (Zamir et al., 2022). Therefore, it is crucial to identify markers according to the pathogenesis of POTS to predict which children with POTS will respond to metoprolol and guide the application of metoprolol. The reported pathogenesis of POTS includes autonomic dysfunction (Shannon et al., 2000), abnormal blood vessel dilation (Liao et al., 2013), and hypovolemia (Zhang et al., 2012). Moreover, metoprolol is more effective in treating children with high-adrenergic status (Thieben et al., 2007). Zhang et al. (2014) found that orthostatic plasma noradrenaline could be used to predict the efficacy of using metoprolol to treat POTS. When noradrenaline levels were > 3.59 pg./mL, metoprolol was predicted to be effective, with a sensitivity of 76.9% and a specificity of 91.7% (Zhang et al., 2014). However, as previously mentioned, various physiological and pathological factors affect the plasma noradrenaline level. To identify patients suitable to receive metoprolol at the time of the initial diagnosis, Zhao et al. (2014) found that copeptin levels <10.225 pmol/L (sensitivity: 90.5%; specificity:78.6%) were useful, and Lin et al. (2015) found that CNP > 32.55 pg/mL (sensitivity: 95.8%; specificity: 70%) were applicable. However, assessing copeptin and CNP levels requires blood collection procedures. Wang et al. (2019) demonstrated that the HR variability (TR index <33.7 and SDNN index <79.0 ms) could be used as a predictor in this field, but these indexes are not intuitive. Skin sympathetic activity can be measured directly by microneurography, which can reflect the dynamic changes of sympathetic activity. However, microneurography, which records sympathetic activity by inserting thin tungsten needle electrodes into the nerve, is invasive and technically difficult; therefore, it is not the most feasible and convenient method for daily monitoring (Kusayama et al., 2020; Xing et al., 2022). A new method for real-time assessment of the autonomic nervous system, called neuECG, has recently been reported, which noninvasively records both skin sympathetic activity and ECG. neuECG has the advantages of being non-invasive, convenient, and accurate and having good signal recording performance. However, the electrical activity measured by neuECG on the skin surface comes from the heart, muscle, or nerve structure, and there is a lot of interference. In addition, neuECG is mainly used in healthy people and patients without cardiovascular disease, and its requirements for equipment are relatively high, which is not conducive to clinical application (Kusayama et al., 2020; Xing et al., 2022). Therefore, when managing children with POTS, it is essential to further explore stable, comfortable, and convenient predictors of the metoprolol treatment outcomes.

Poincaré plots are usually used in Holter ECG analysis, as they can display all the RR intervals of the 24-h Holter ECG data in one scatterplot and intuitively reflect autonomic activity using graphical characteristics. The T of the graph indicates the instant change in the cardiac cycle and is positively correlated with vagal nerve tension (Chaidas et al., 2014). Although the clinical value of L is not fully understood, some scholars consider it to indicate the sympathetic tone (Naranjo Orellana et al., 2015). The L/T reflects the interaction between sympathetic and vagal activities (Naranjo Orellana et al., 2015). Studies have shown that, with the augmentation of sympathetic function, the shape of the Poincaré plot becomes almost baseball bat-like, with the increase in L/T ratio (Toichi et al., 1997). Together, these factors form the theoretical basis for using a Poincaré plot to help predict the treatment outcomes of metoprolol.

In this study, we explored the application of Poincaré plots to identify children with POTS for whom metoprolol treatment is suitable. The results revealed that the L and T of pediatric patients who responded to metoprolol were visibly smaller than those who did not, and the L/T of children who responded was visibly greater than those who did not. The results indicated that the sympathetic activity of responders to metoprolol before treatment was greater than that of non-responders, suggesting that metoprolol may inhibit excessive sympathetic excitation by antagonizing β1-receptors thus improving the patients’ symptoms. Moreover, the results showed that a T < 573.9 ms combined with an L/T > 2.9 had high sensitivity, specificity, and accuracy in prejudging the treatment outcome of metoprolol for pediatric patients with POTS, reaching 85.0, 82.6, and 84.1%, respectively.

Wang et al. (2019) have shown that when the triangular index and SDNNI were ≤ 33.7 ms and ≤ 79.0 ms, respectively, a good outcome of metoprolol treatment for pediatric POTS can be prejudged (sensitivity: 85.3%; specificity: 81.8%; accuracy: 84.4%). HR variability indexes and Poincaré plots are tools for assessing autonomic function. We combined HR variability indexes and Poincaré plot indexes for prediction to further enhance efficiency. The results showed that the parameters from the Poincaré plot were better than the HR variability indexes, according to the series–parallel analysis in our study. We found that the HR variability indexes rMSSD, pNN50, HF, and TP of children who responded were distinctly lower than those who did not respond, similar to a previous finding (Wang et al., 2019). Time-domain indexes of HR variability (e.g., rMSSD and pNN50) can reflect changes in vagal tone, which is believed to be the result of vagal excitation (Wang et al., 2019). In our study, ROC analysis indicated that the area under the curve of the parameters from the Poincaré plot was greater than the HR variability indexes when these parameters were measured in the same population. This indicates that the parameters of the Poincaré plot were better predictors than the HR variability indexes in predicting the treatment outcome of metoprolol. Finally, the Poincaré plot has the advantage of visualization, which is more intuitive than previous predictors. Graphically, the Poincaré plot of a “baseball bat shape” (T < 573.9 ms combined with L/T > 2.9) is more likely to indicate that the patient will respond well to the metoprolol treatment, while the Poincaré plot of a “tennis racket shape” (T ≥ 573.9 ms and L/T ≤ 2.9) is more likely to indicate a poor response. Therefore, our results showed that the Poincaré plot of children with POTS before treatment can be used as a noninvasive, stable, efficient, and intuitive indicator of the efficacy of metoprolol in children with POTS.

Our study had some limitations, including the single-center retrospective design, small number of samples, relatively basic graphic measurement methods, and lack of validation studies to verify the predictive efficiency of the indicators. In addition, some other methods can calculate HR variability, such as some nonlinear dynamic indicators analyzed by entropy measures (Castiglioni et al., 2023). The role of other indicators for HR variability in predicting the efficacy of metoprolol should be discussed in the future. However, this study innovatively demonstrates that the noninvasive, stable, efficient, and intuitive parameters of the Poincaré plot can be used for children with POTS who may be suitable to undergo metoprolol treatment, which facilitates the individualized treatment of children with POTS. Further multicenter, prospective, large-sample, and external validation studies should be conducted to validate our results through more accurate graphic analysis techniques to promote better individualized treatments for children with POTS.

In conclusion, this study innovatively confirmed the predictive value of the Poincaré plot for treatment outcomes of using metoprolol for pediatric POTS. A T < 573.9 ms combined with an L/T > 2.9 (baseball bat shape) can help predict good outcomes for metoprolol treatment of pediatric POTS.

## Data availability statement

The original contributions presented in the study are included in the article/Supplementary material, further inquiries can be directed to the corresponding authors.

## Ethics statement

The studies involving humans were approved by Ethics Committee of Peking University First Hospital (2021 [150]). The studies were conducted in accordance with the local legislation and institutional requirements. Informed consent for participation in this study was provided by the participants’ legal guardians/next of kin.

## Author contributions

PY: Data curation, Investigation, Methodology, Software, Writing – original draft, Writing – review & editing. ZL: Data curation, Formal analysis, Investigation, Software, Writing – review & editing. YW: Data curation, Formal analysis, Investigation, Software, Writing – review & editing. CZ: Data curation, Formal analysis, Investigation, Software, Writing – review & editing. HJ: Funding acquisition, Project administration, Resources, Supervision, Writing – review & editing. JD: Funding acquisition, Project administration, Resources, Supervision, Writing – review & editing. YH: Conceptualization, Funding acquisition, Methodology, Project administration, Resources, Supervision, Writing – review & editing. YL: Conceptualization, Funding acquisition, Methodology, Project administration, Resources, Supervision, Writing – review & editing.

## References

[ref1] Boris J. R. (2018). Postural orthostatic tachycardia syndrome in children and adolescents. Auton. Neurosci. 215, 97–101. doi: 10.1016/j.autneu.2018.05.004, PMID: 29778304

[ref2] Brennan M. Palaniswami M. Kamen P. (2002). Poincaré plot interpretation using a physiological model of HRV based on a network of oscillators. Am. J. Physiol. Heart Circ. Physiol. 283, H1873–H1886. doi: 10.1152/ajpheart.00405.2000, PMID: 12384465

[ref3] Bryarly M. Phillips L. T. Fu Q. Vernino S. Levine B. D. (2019). Postural orthostatic tachycardia syndrome: JACC focus seminar. J. Am. Coll. Cardiol. 73, 1207–1228. doi: 10.1016/j.jacc.2018.11.059 30871704

[ref4] Carrasco S. Gaitán M. J. González R. Yánez O. (2001). Correlation among Poincaré plot indexes and time and frequency domain measures of heart rate variability. J. Med. Eng. Technol. 25, 240–248. doi: 10.1080/03091900110086651 11780765

[ref5] Castiglioni P. Merati G. Parati G. Faini A. (2023). Sample, fuzzy and distribution entropies of heart rate variability: what do they tell us on cardiovascular complexity? Entropy (Basel, Switzerland) 25:281. doi: 10.3390/e25020281, PMID: 36832650 PMC9954876

[ref6] Chaidas K. Tsaoussoglou M. Theodorou E. Lianou L. Chrousos G. Kaditis A. G. (2014). Poincaré plot width, morning urine norepinephrine levels, and autonomic imbalance in children with obstructive sleep apnea. Pediatr. Neurol. 51, 246–251. doi: 10.1016/j.pediatrneurol.2014.05.003, PMID: 25079573

[ref7] Chen G. Du J. Jin H. Huang Y. (2020). Postural tachycardia syndrome in children and adolescents: pathophysiology and clinical management. Front. Pediatr. 8:474. doi: 10.3389/fped.2020.00474 32974246 PMC7468430

[ref8] Chen L. Wang L. Sun J. Qin J. Tang C. Jin H.. (2011). Midodrine hydrochloride is effective in the treatment of children with postural orthostatic tachycardia syndrome. Circ. J. 75, 927–931. doi: 10.1253/circj.CJ-10-0514, PMID: 21301135

[ref9] Copie X. Pousset F. Lechat P. Jaillon P. Guize L. Le Heuzey J. Y. (1996). Effects of beta-blockade with bisoprolol on heart rate variability in advanced heart failure: analysis of scatterplots of R-R intervals at selected heart rates. Am. Heart J. 132, 369–375. doi: 10.1016/S0002-8703(96)90435-4, PMID: 8701900

[ref10] Keeley E. C. Lange R. A. Hillis L. D. Joglar J. A. Page R. L. (1997). Correlation between time-domain measures of heart rate variability and scatterplots in patients with healed myocardial infarcts and the influence of metoprolol. Am. J. Cardiol. 79, 412–414. doi: 10.1016/S0002-9149(96)00777-1, PMID: 9052341

[ref11] Kusayama T. Wong J. Liu X. He W. Doytchinova A. Robinson E. A.. (2020). Simultaneous noninvasive recording of electrocardiogram and skin sympathetic nerve activity (neuECG). Nat. Protoc. 15, 1853–1877. doi: 10.1038/s41596-020-0316-6, PMID: 32313253

[ref12] Lai C. C. Fischer P. R. Brands C. K. Fisher J. L. Porter C. B. Driscoll S. W.. (2009). Outcomes in adolescents with postural orthostatic tachycardia syndrome treated with midodrine and beta-blockers. Pacing Clin. Electrophysiol. 32, 234–238. doi: 10.1111/j.1540-8159.2008.02207.x, PMID: 19170913

[ref13] Li H. Wang Y. Liu P. Chen Y. Feng X. Tang C.. (2016). Body mass index (BMI) is associated with the therapeutic response to oral rehydration solution in children with postural tachycardia syndrome. Pediatr. Cardiol. 37, 1313–1318. doi: 10.1007/s00246-016-1436-1, PMID: 27350278

[ref14] Liao Y. Yang J. Zhang F. Chen S. Liu X. Zhang Q.. (2013). Flow-mediated vasodilation as a predictor of therapeutic response to midodrine hydrochloride in children with postural orthostatic tachycardia syndrome. Am. J. Cardiol. 112, 816–820. doi: 10.1016/j.amjcard.2013.05.008, PMID: 23735645

[ref15] Lin J. Han Z. Li H. Chen S. Y. Li X. Liu P.. (2015). Plasma C-type natriuretic peptide as a predictor for therapeutic response to metoprolol in children with postural tachycardia syndrome. PLoS One 10:e0121913. doi: 10.1371/journal.pone.0121913, PMID: 25811760 PMC4374798

[ref16] Lin J. Wang Y. Zhang Q. Liao Y. Qi J. Liu P.. (2016). Underlying disease spectrum of syncope in children and adolescents in the past 30 years and health economic analysis: a single center report. Chin. J. Prac. Pediatr. 31, 350–355. doi: 10.7504/ek2016050609

[ref17] Medow M. S. Kothari M. L. Goetz A. M. O’Donnell-Smith M. B. Terilli C. Stewart J. M. (2017). Decreasing cerebral oxygen consumption during upright tilt in vasovagal syncope. Physiol. Rep. 5:13286. doi: 10.14814/phy2.13286, PMID: 28554964 PMC5449565

[ref18] Naranjo Orellana J. de la Cruz Torres B. Sarabia Cachadiña E. de Hoyo M. Domínguez Cobo S. (2015). Two new indexes for the assessment of autonomic balance in elite soccer players. Int. J. Sports Physiol. Perform. 10, 452–457. doi: 10.1123/ijspp.2014-0235, PMID: 25364865

[ref19] Shannon J. R. Flattem N. L. Jordan J. Jacob G. Black B. K. Biaggioni I.. (2000). Orthostatic intolerance and tachycardia associated with norepinephrine-transporter deficiency. N. Engl. J. Med. 342, 541–549. doi: 10.1056/NEJM200002243420803, PMID: 10684912

[ref20] Spahic J. M. Ricci F. Aung N. Axelsson J. Melander O. Sutton R.. (2019). Proconvertase Furin is downregulated in postural orthostatic tachycardia syndrome. Front. Neurosci. 13:301. doi: 10.3389/fnins.2019.00301 31001074 PMC6455076

[ref21] Stewart J. M. (2002). Orthostatic intolerance in pediatrics. J. Pediatr. 140, 404–411. doi: 10.1067/mpd.2002.122727, PMID: 12006953

[ref22] Stewart J. M. (2013). Common syndromes of orthostatic intolerance. Pediatrics 131, 968–980. doi: 10.1542/peds.2012-2610, PMID: 23569093 PMC3639459

[ref23] Thieben M. J. Sandroni P. Sletten D. M. Benrud-Larson L. M. Fealey R. D. Vernino S.. (2007). Postural orthostatic tachycardia syndrome: the mayo clinic experience. Mayo Clin. Proc. 82, 308–313. doi: 10.4065/82.3.308, PMID: 17352367

[ref24] Toichi M. Sugiura T. Murai T. Sengoku A. (1997). A new method of assessing cardiac autonomic function and its comparison with spectral analysis and coefficient of variation of R-R interval. J. Auton. Nerv. Syst. 62, 79–84. doi: 10.1016/s0165-1838(96)00112-9 9021653

[ref25] Ueda T. Nakatsu T. Yamane S. Kurazono S. Murakami T. Mashima K.. (2002). Correlation of Lorenz scatterplots with frequency domain heart rate variability. Clin. Exp. Hypertens. 24, 11–21. doi: 10.1081/CEH-100108711, PMID: 11848164

[ref26] Wang C. Li Y. Liao Y. Tian H. Huang M. Dong X.. (2018). 2018 Chinese pediatric cardiology society (CPCS) guideline for diagnosis and treatment of syncope in children and adolescents. Sci. Bull. 63, 1558–1564. doi: 10.1016/j.scib.2018.09.019, PMID: 36751076

[ref27] Wang Y. Sun Y. Zhang Q. Zhang C. Liu P. Wang Y.. (2021). Baseline corrected QT interval dispersion is useful to predict effectiveness of metoprolol on pediatric postural tachycardia syndrome. Front. Cardiovasc. Med. 8:808512. doi: 10.3389/fcvm.2021.808512, PMID: 35127870 PMC8812810

[ref28] Wang Y. Zhang C. Chen S. Liu P. Wang Y. Tang C.. (2019). Heart rate variability predicts therapeutic response to metoprolol in children with postural tachycardia syndrome. Front. Neurosci. 13:1214. doi: 10.3389/fnins.2019.01214 31780890 PMC6861190

[ref29] Wells R. Spurrier A. J. Linz D. Gallagher C. Mahajan R. Sanders P.. (2018). Postural tachycardia syndrome: current perspectives. Vasc. Health Risk Manag. 14, 1–11. doi: 10.2147/VHRM.S127393, PMID: 29343965 PMC5749569

[ref30] Xing Y. T. Zhang Y. K. Yang C. X. Li J. Q. Li Y. W. Cui C.. (2022). Design and evaluation of an autonomic nerve monitoring system based on skin sympathetic nerve activity. Biomed. Signal Proc. Control 76:103681. doi: 10.1016/j.bspc.2022.103681

[ref31] Yuan P. Li X. Tao C. Du X. Zhang C. Du J.. (2022a). Poincaré plot can be a useful tool to select potential responders to metoprolol therapy in children with vasovagal syncope. Int. J. Gen. Med. 15, 2681–2693. doi: 10.2147/ijgm.s352928 35300141 PMC8922042

[ref32] Yuan P. Lian Z. Wang Y. Wang Y. Zhang C. Du J.. (2022b). Poincaré plot is useful for distinguishing vasovagal syncope from postural tachycardia syndrome in children. Front. Pediatr. 10:758100. doi: 10.3389/fped.2022.758100 35372154 PMC8965582

[ref33] Zamir A. Hussain I. Ur Rehman A. Ashraf W. Imran I. Saeed H.. (2022). Clinical pharmacokinetics of metoprolol: a systematic review. Clin. Pharmacokinet. 61, 1095–1114. doi: 10.1007/s40262-022-01145-y, PMID: 35764772

[ref34] Zhang Q. Chen X. Li J. Du J. (2014). Orthostatic plasma norepinephrine level as a predictor for therapeutic response to metoprolol in children with postural tachycardia syndrome. J. Transl. Med. 12:249. doi: 10.1186/s12967-014-0249-3 25204388 PMC4177336

[ref35] Zhang Q. Liao Y. Tang C. Du J. Jin H. (2012). Twenty-four-hour urinary sodium excretion and postural orthostatic tachycardia syndrome. J. Pediatr. 161, 281–284. doi: 10.1016/j.jpeds.2012.01.054, PMID: 22424949

[ref36] Zhao J. Du S. Yang J. Lin J. Tang C. Du J.. (2014). Usefulness of plasma copeptin as a biomarker to predict the therapeutic effectiveness of metoprolol for postural tachycardia syndrome in children. Am. J. Cardiol. 114, 601–605. doi: 10.1016/j.amjcard.2014.05.039, PMID: 24996552

