# Supplementary material for: Poincaré plot can help predict the curative effect of metoprolol for pediatric postural orthostatic tachycardia syndrome
Source: Front Neurosci. 2023 Nov 14;17:1280172. doi: 10.3389/fnins.2023.1280172 (PMC10682374; doi:10.3389/fnins.2023.1280172)
Supplement: Supplementary file 1 [file Data_Sheet_1.doc]

Supplementary materials

# The method and results of stability and repeatability test of the Poincaré plots measurements:

# The longitudinal axis and transverse axis of Poincaré plots of all included children were measured by a dedicated investigator. Prior to this, the stability and repeatability of the measurements were tested. In the stability test, the dedicated investigator performed two measurements on the Poincaré plots from 15 subjects with an interval of one week, and then the values of the two measurements were compared (Supplemental Table 1). In the repeatability test, two investigators (including the dedicated one) measured the Poincaré plots from 15 subjects independently, and then the measured values of Poincaré plots from each subject were compared (Supplemental Table 2). The stability and repeatability of the Poincaré plots measurements were tested by paired *t* test.

**Supplemental Table 1 Results of measurement (two trials) of the graphic parameters of poincaré plot in 15 subjects**

| Subject number | L, ms | |  | T, ms | |  | L/T | |  |
| --- | --- | --- | --- | --- | --- | --- | --- | --- | --- |
|  | First time | Second time |  | First time | Second time |  | First time | Second time |  |
| Subject 1 | 1188.77 | 1178.48 |  | 399.64 | 399.64 |  | 2.97 | 2.95 |  |
| Subject 2 | 1803.62 | 1803.62 |  | 748.12 | 748.12 |  | 2.41 | 2.41 |  |
| Subject 3 | 1516.67 | 1526.96 |  | 348.41 | 348.41 |  | 4.35 | 4.38 |  |
| Subject 4 | 1434.71 | 1434.71 |  | 256.23 | 266.45 |  | 5.60 | 5.38 |  |
| Subject 5 | 1803.62 | 1813.91 |  | 758.33 | 758.33 |  | 2.38 | 2.39 |  |
| Subject 6 | 1362.97 | 1352.75 |  | 348.41 | 348.41 |  | 3.91 | 3.88 |  |
| Subject 7 | 1711.38 | 1711.38 |  | 830.07 | 840.36 |  | 2.06 | 2.04 |  |
| Subject 8 | 1506.45 | 1506.45 |  | 327.90 | 297.17 |  | 4.59 | 5.07 |  |
| Subject 9 | 1209.28 | 1198.99 |  | 379.20 | 368.91 |  | 3.19 | 3.25 |  |
| Subject 10 | 1332.25 | 1342.46 |  | 245.94 | 235.72 |  | 5.42 | 5.70 |  |
| Subject 11 | 1393.70 | 1383.48 |  | 225.43 | 225.43 |  | 6.18 | 6.14 |  |
| Subject 12 | 1762.61 | 1772.90 |  | 532.90 | 522.61 |  | 3.31 | 3.39 |  |
| Subject 13 | 1455.22 | 1455.2 |  | 286.96 | 286.96 |  | 5.07 | 5.07 |  |
| Subject 14 | 1947.10 | 1947.10 |  | 912.10 | 901.81 |  | 2.13 | 2.16 |  |
| Subject 15 | 737.83 | 737.83 |  | 143.48 | 153.70 |  | 5.14 | 4.80 |  |
| Paired samples | r = 1.000 | |  | r = 0.999 | |  | r = 0.991 | |  |
| Correlation | *P* < 0.001 | |  | *P* < 0.001 | |  | *P* < 0.001 | |  |
| Paired *t* test | *P* = 0.998 | |  | *P* = 0.333 | |  | *P* = 0.697 | |  |

**Abbreviations:** L, longitudinal axis; ms, millisecond; T, transverse axis; L/T, the ratio of longitudinal axis value to transverse axis value.

**Supplemental Table 2 Reproductivity of graphic parameters of poincaré plot measurement between different operators**

| Subject number | L, ms | |  | T, ms | |  | L/T | |  |
| --- | --- | --- | --- | --- | --- | --- | --- | --- | --- |
|  | Operator 1 | Operator 2 |  | Operator 1 | Operator 2 |  | Operator 1 | Operator 2 |  |
| Subject 1 | 1188.77 | 1198.99 |  | 399.64 | 409.93 |  | 2.97 | 2.92 |  |
| Subject 2 | 1803.62 | 1793.41 |  | 748.12 | 758.33 |  | 2.41 | 2.36 |  |
| Subject 3 | 1516.67 | 1516.67 |  | 348.41 | 348.41 |  | 4.35 | 4.35 |  |
| Subject 4 | 1434.71 | 1434.71 |  | 256.23 | 256.23 |  | 5.60 | 5.60 |  |
| Subject 5 | 1803.62 | 1803.62 |  | 758.33 | 748.12 |  | 2.38 | 2.41 |  |
| Subject 6 | 1362.97 | 1362.97 |  | 348.41 | 358.70 |  | 3.91 | 3.80 |  |
| Subject 7 | 1711.38 | 1721.67 |  | 830.07 | 819.86 |  | 2.06 | 2.10 |  |
| Subject 8 | 1506.45 | 1496.23 |  | 327.90 | 317.68 |  | 4.59 | 4.71 |  |
| Subject 9 | 1209.28 | 1209.28 |  | 379.20 | 358.70 |  | 3.19 | 3.37 |  |
| Subject 10 | 1332.25 | 1321.96 |  | 245.94 | 245.94 |  | 5.42 | 5.38 |  |
| Subject 11 | 1393.70 | 1393.70 |  | 225.43 | 225.43 |  | 6.18 | 6.18 |  |
| Subject 12 | 1762.61 | 1762.61 |  | 532.90 | 532.90 |  | 3.31 | 3.31 |  |
| Subject 13 | 1455.22 | 1444.93 |  | 286.96 | 286.96 |  | 5.07 | 5.04 |  |
| Subject 14 | 1947.10 | 1936.88 |  | 912.10 | 912.10 |  | 2.13 | 2.12 |  |
| Subject 15 | 737.83 | 737.83 |  | 143.48 | 143.48 |  | 5.14 | 5.14 |  |
| Paired samples | r = 1.000 | |  | r = 0.999 | |  | r = 0.999 | |  |
| Correlation | *P* < 0.001 | |  | *P* < 0.001 | |  | *P* < 0.001 | |  |
| Paired *t* test | *P* = 0.271 | |  | *P* = 0.548 | |  | *P* = 0.796 | |  |

**Abbreviations:** L, longitudinal axis; ms, millisecond; T, transverse axis; L/T, the ratio of longitudinal axis value to transverse axis value.

**Supplemental Table 3 Predictive value of time-domain indices and frequency-domain indices of HR variability**

**on the efficacy of metoprolol**

| Variables | AUC | *P* | 95% CI | Cut-off value | Sensitivity (%) | Specificity (%) |
| --- | --- | --- | --- | --- | --- | --- |
| L | 0.761 | 0.001 | 0.630–0.893 | 1680.7 ms | 77.5 | 73.9 |
| T | 0.866 | < 0.001 | 0.764–0.969 | 573.9 ms | 87.5 | 78.3 |
| L/T | 0.857 | < 0.001 | 0.757–0.956 | 2.9 | 85.0 | 73.9 |
| rMSSD | 0.690 | 0.013 | 0.544–0.835 | 59.5 ms | 85.0 | 52.2 |
| pNN50 | 0.668 | 0.027 | 0.523–0.814 | 27.5 ms | 85.0 | 47.8 |
| HF | 0.688 | 0.014 | 0.532–0.844 | 1144.0 ms^2^ | 90.0 | 56.5 |
| TP | 0.676 | 0.021 | 0.520–0.832 | 4740.7 ms^2^ | 95.0 | 47.8 |
| LF/HF | 0.657 | 0.039 | 0.509–0.806 | 1.1 | 92.5 | 43.5 |
| SDNN | 0.654 | 0.043 | 0.510–0.799 | 152.5 | 72.5 | 65.2 |
| Triangular index | 0.642 | 0.061 | 0.498–0.787 | 33.5 | 80.0 | 52.2 |
| SDNNI | 0.654 | 0.043 | 0.504–0.804 | 82.0 | 87.5 | 52.2 |
| SDANN | 0.615 | 0.132 | 0.474–0.755 | 132.5 | 60.0 | 73.9 |
| ULF | 0.623 | 0.107 | 0.470–0.775 | 16351.5 | 67.5 | 65.2 |
| VLF | 0.637 | 0.072 | 0.485–0.789 | 3380.3 | 87.5 | 43.5 |
| LF | 0.648 | 0.052 | 0.490–0.806 | 1456.2 | 92.5 | 47.8 |

**Abbreviations:** HR, heart rate; SDNN, standard deviation of normal-to-normal intervals; SDNNI, standard deviation of the averages of NN intervals; rMSSD, root mean square of the successive differences; pNN50, percentage of adjacent NN intervals that differed by > 50 ms; SDANN, standard deviation of the averages of normal-to-normal intervals in all 5-min segments of the entire recording; ULF, ultralow frequency; VLF, very low frequency; LF, low frequency; HF, high frequency; TP, total power; L, longitudinal axis; T, transverse axis.
